# Development of a Novel Transparent Flexible Capacitive Micromachined Ultrasonic Transducer

**DOI:** 10.3390/s17061443

**Published:** 2017-06-20

**Authors:** Da-Chen Pang, Cheng-Min Chang

**Affiliations:** Department of Mechanical Engineering, National Kaohsiung University of Applied Sciences, 415 Jian Gong Rd., Sanmin Dist., Kaohsiung 80778, Taiwan; v6302001@gmail.com

**Keywords:** capacitive micromachined ultrasonic transducer, flexible transducer, silver nanowire transparent electrode, SU-8, human-machine interface

## Abstract

This paper presents the world’s first transparent flexible capacitive micromachined ultrasonic transducer (CMUT) that was fabricated through a roll-lamination technique. This polymer-based CMUT has advantages of transparency, flexibility, and non-contacting detection which provide unique functions in display panel applications. Comprising an indium tin oxide-polyethylene terephthalate (ITO-PET) substrate, SU-8 sidewall and vibrating membranes, and silver nanowire transparent electrode, the transducer has visible-light transmittance exceeding 80% and can operate on curved surfaces with a 40 mm radius of curvature. Unlike the traditional silicon-based high temperature process, the CMUT can be fabricated on a flexible substrate at a temperature below 100 °C to reduce residual stress introduced at high temperature. The CMUT on the curved surfaces can detect a flat target and finger at distances up to 50 mm and 40 mm, respectively. The transparent flexible CMUT provides a better human-machine interface than existing touch panels because it can be integrated with a display panel for non-contacting control in a health conscious environment and the flexible feature is critical for curved display and wearable electronics.

## 1. Introduction

In 1989, Hohm and Hess [1] presented the first capacitive ultrasonic transducer. The earlier transducers were fabricated using anisotropic etching of silicon backplates [1,2,3]. In 1994, Haller and Khuri-Yakub [4] developed a capacitive micromachined ultrasonic transducer (CMUT) using sacrificial-layer technology. The Khuri-Yakub group at Stanford University had also proposed fabrication improvements and CMUT applications in the literature [5,6,7,8,9]. In 2003, Huang et al. [10] fabricated a CMUT using wafer bonding technology to ensure fewer process steps and better product quality compared with the sacrificial-layer processes. The wafer bonding technology including anodic bonding, fusion bonding, and adhesive bonding has been extensively applied in the fabrication of CMUTs [11,12,13,14,15,16,17,18]. The opaqueness of silicon wafers to visible light causes non-transparent properties in silicon-based CMUTs. Since infrared light can pass through silicon, Chen et al. [19] presented an infrared-transparent CMUT for photoacoustic imaging in 2012.

In 2006, Chang et al. [20] pioneered a polymer-based CMUT using sacrificial-layer techniques and later fabricated on a polymer substrate so the CMUT was flexible [21]. The CMUTs on an ultrathin silicon wafer also feature bending characteristics [22]. In 2008, Zhuang et al. [23] etched trenches into silicon wafers and filled them with polydimethylsiloxane (PDMS) to fabricate flexible CMUT arrays. In 2008, Abgrall et al. [24] applied the lamination technique for minimizing residual stress to fabricate SU-8 bonding structures at low pressure and temperature. In 2012, Shi et al. [25] used Polydimethylsiloxane (PDMS) and bonding technology to fabricate a stretchable CMUT but the CMUT was not transparent due to the metal electrodes. In 2015, Li et al. [26] fabricated a polymer-based CMUT using photo benzocyclobutene and bonding technology but the membrane was made of silicon nitride and the process temperature was up to 250 °C. In 2015, Bui et al. [27] used a polymer-based CMUT to measure surface roughness. All the polymer-based CMUTs above were non-transparent.

**Figure 1:**
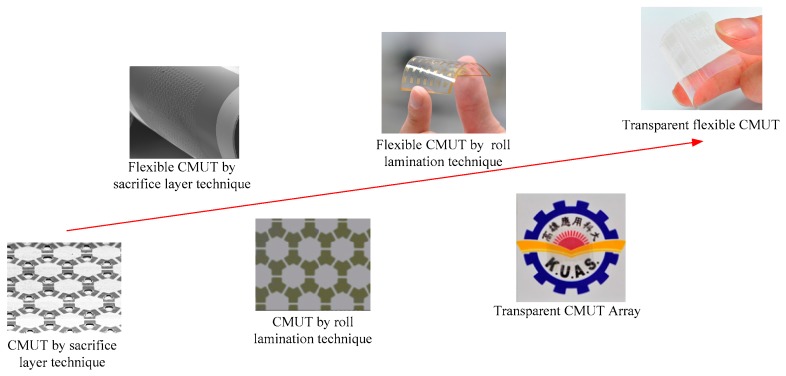
CMUT research results from our group.

The purpose of this research is to develop a novel fabrication process for a transparent flexible CMUT so the CMUT can be applied in a display panel for finger hovering that provides a more advanced human-machine interface than the existing touch panel. A new roll-lamination fabrication technique is proposed for the mass production of CMUTs at low temperature. The roll-lamination method minimizes the residual stress introduced at high temperature if a bonding process is used. This fabrication method is also simpler than the sacrificial-layer technique. Three different transparent electrodes, indium tin oxide (ITO), aluminum-doped zinc oxide (AZO), and silver nanowire, are fabricated and tested in our CMUTs. The transparent electrodes on the vibrating membranes must survive ultrasonic vibration under long term operation. The performance characteristics of the transparent flexible CMUT are tested on flat and curved surfaces. The proposed CMUT can be easily integrated with display panels and lighting systems for non-contacting sensing and control in the future. Figure 1 shows the research progress of our group over the years.

## 2. CMUT Design

**Figure 2:**
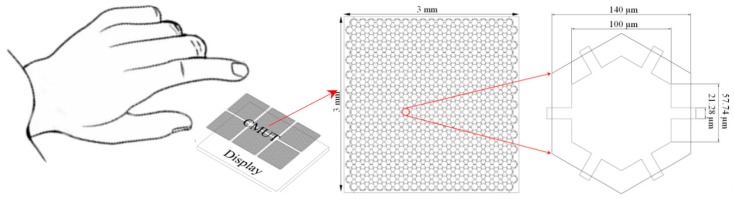
Schematic Diagram of finger hovering using a transparent capacitive micromachined ultrasonic transducer (CMUT).

**Figure 3:**
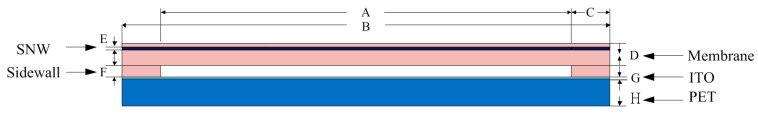
Cross-sectional view of a transparent CMUT.

The proposed transparent CMUT has a surface area of 3 mm × 3 mm and comprises 416 hexagon-inscribed vibrating membranes with a diameter of 140 µm, as illustrated in Figure 2. The ITO-PET substrate was 125 µm in thickness, the sidewall was 10 µm in width, the cavity was 2 µm in depth, and the vibrating membranes, which sandwiched a 0.2 µm thick silver nanowire (SNW) transparent electrode, were 5 µm in thickness as shown in Figure 3. The design dimensions are listed in Table 1. A total of 72 CMUTs with an overall size of 8 cm × 6 cm were fabricated on a 4-inch silicon wafer.

## 3. Fabrication

**Figure 4:**
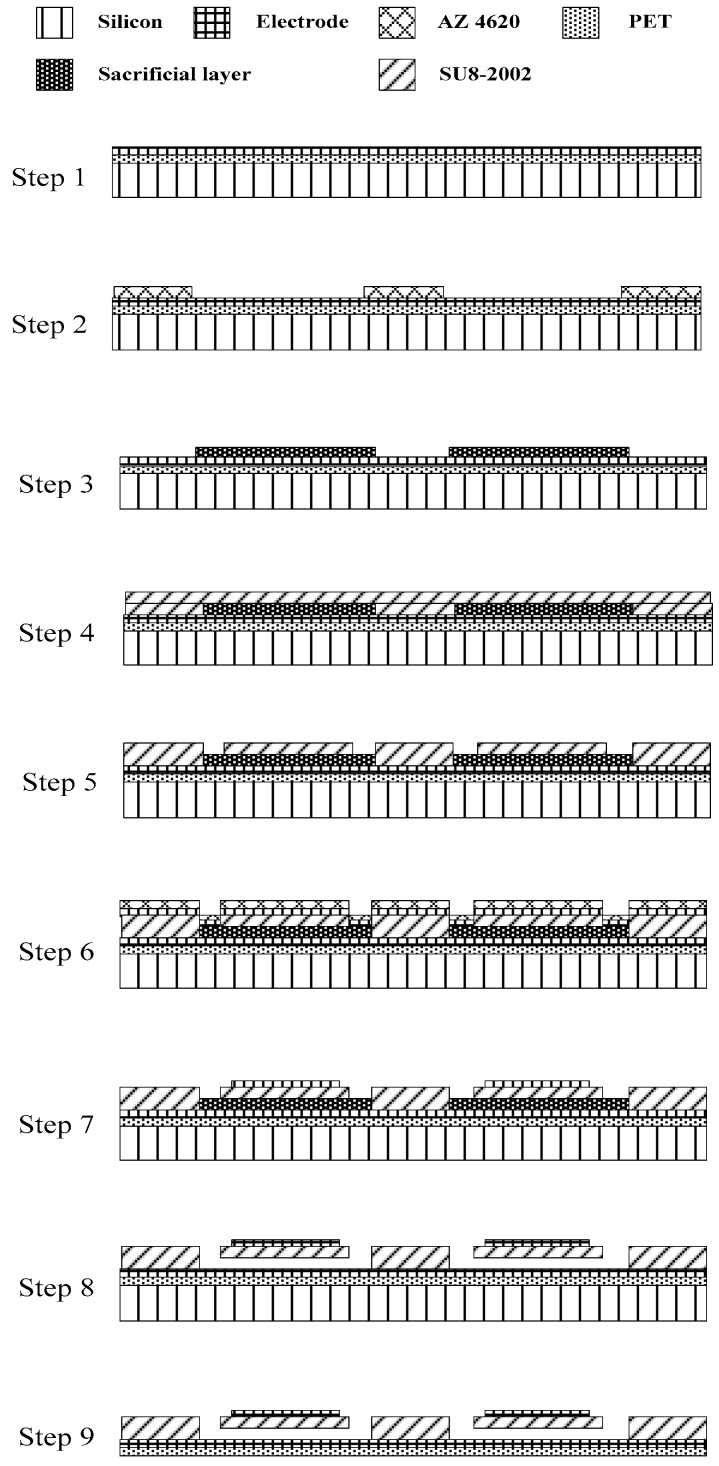
CMUT made by the sacrificial-layer fabrication technique.

The fabrication of the transparent flexible CMUT was built on a polymer-based CMUT using sacrificial-layer techniques developed earlier. The polymer-based CMUT applied a PET substrate and SU-8 structure and membrane. It was not transparent because platinum and gold were used for the electrodes. The sacrificial-layer fabrication procedure of the polymer-based CMUT, depicted in Figure 4, is described as follows:

1. Paste a PET flexible substrate onto a silicon wafer and sputter a 0.3 µm thick platinum electrode.
2. Pattern a 2 µm thick AZ4620 photoresist to protect the sidewall area, followed by soft baking at 95 °C for 2 min.
3. Electroform 2 µm thick copper as a sacrificial layer. Remove the AZ4620 photoresist.
4. Pattern a SU-8 2002 photoresist to form a sidewall and vibrating membrane. Perform a soft bake at 65 °C for 4 min and 95 °C for 4 min, and then post exposure bake at 65 °C for 2 min and 95 °C for 3 min.
5. Develop the SU-8 2002 photoresist to yield etching holes. Perform a hard bake at 95 °C for 5 min.
6. Deposit 0.3 µm thick gold to yield the top electrode layer.
7. Pattern-etch the top electrode using the AZ4620 photoresist and potassium iodine.
8. Remove the copper sacrificial layer to release the vibrating membranes and cavities.
9. Remove the silicon wafer to complete the CMUT fabrication.

**Figure 5:**
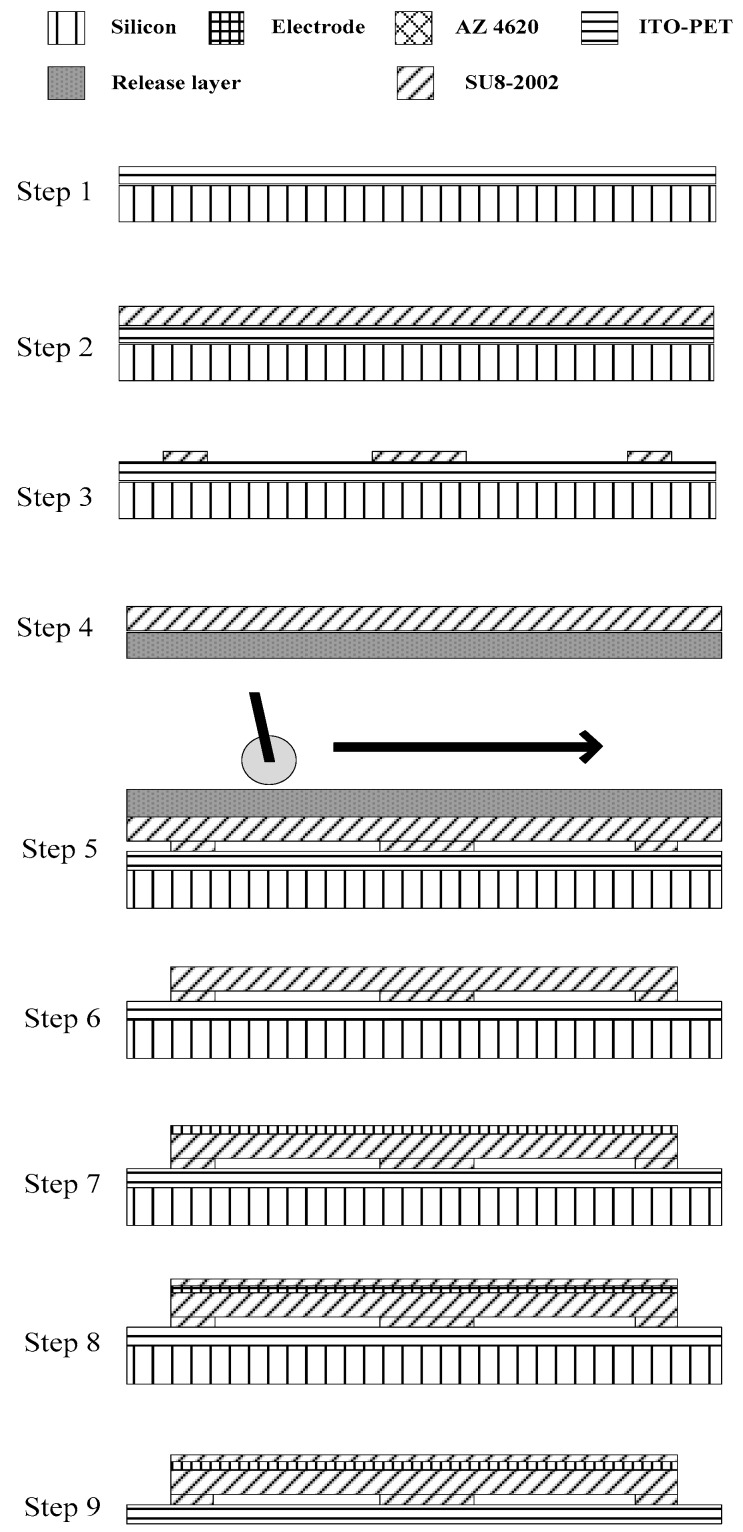
Transparent CMUT made by the roll-lamination fabrication technique.

This research developed a new roll-lamination fabrication procedure for the transparent flexible CMUT without time and cost for the electroforming process compared with the sacrificial-layer technique. The CMUT exhibited transparency and flexibility by employing an ITO-PET substrate, SU 8 structure and membrane, and silver nanowire electrode. The new fabrication method applied the roll-lamination technique and dip coating process so the process temperatures were below 100 °C. The fabrication procedure of the transparent flexible CMUT, illustrated in Figure 5, is described as follows:

1. Paste a 125 µm thick ITO-PET substrate onto a silicon wafer.
2. Spin coat a 2 µm thick SU-8 2002 photoresist onto the ITO-PET substrate, followed by soft bake at 65 °C for 3 min, 95 °C for 3 min, and then 65 °C for 3 min.
3. Pattern the SU-8 2002 photoresist to form a sidewall. Perform a post exposure bake at 65 °C for 3 min, 95 °C for 3 min, and 65 °C for 3 min, and then hard bake at 65 °C for 2 min, 95 °C for 4 min, and 65 °C for 2 min.
4. Prepare a 4 µm thick SU-8 2002 photoresist on a PET release layer, followed by soft bake at 65 °C for 3 min and then 95 °C for 2 min.
5. Roll-laminate the PET release layer containing the SU-8 2002 photoresist as vibrating membranes onto the sidewall at an average pressure of 0.35 MPa.
6. Expose the SU-8 2002 photoresist on the PET release layer, followed by post exposure bake at 65 °C for 1 min, 95 °C for 1 min, and 65 °C for 1 min. Remove the PET release layer and develop the vibrating membranes.
7. Prepare a 0.2 µm thick transparent silver nanowire electrode through dip coating.
8. Spin coat a 1 µm thick SU-8 2002 photoresist onto the vibrating membranes, followed by soft bake at 65 °C for 2 min, 95 °C for 2 min, and then 65 °C for 2 min. Pattern the SU-8 2002 photoresist to form a protect layer. Perform a post exposure bake at 65 °C for 2 min, 95 °C for 2 min, and 65 °C for 2 min, and hard bake at 65 °C for 2 min, 95 °C for 3 min, and 65 °C for 2 min.
9. Remove the silicon wafer to complete the transparent CMUT fabrication.

The minimum membrane thicknesses for sacrificial-layer and roll-lamination fabrication procedures are 1 µm and 2 µm, respectively. The limitation of membrane thickness in the roll-lamination fabrication procedure is due to the removal of the PET release layer in step 6. Considering the ratio of membrane thickness over diameter, the sacrificial-layer fabrication procedure achieves the state-of-the-art in micromachining polymer-based CMUTs.

**Figure 6:**
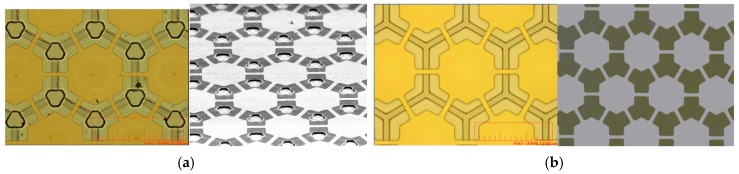
(**a**) CMUT made by the sacrificial-layer technique (left: optical microscope (OM) image; right: scanning electron microscope (SEM) image); (**b**) CMUT made by the roll-lamination technique (left: OM image; right: SEM image).

Figure 6a,b illustrate two CMUTs made respectively by the sacrificial-layer and roll-lamination techniques. Both figures show top electrodes made of gold because the electrode patterns are not clear with silver nanowire. The left and right images were obtained using an optical microscope (OM) and scanning electron microscope (SEM). The etching holes from the sacrificial-layer technique are clearly shown in Figure 6a, whereas there was no hole seen through the roll-lamination technique in Figure 6b.

**Figure 7:**
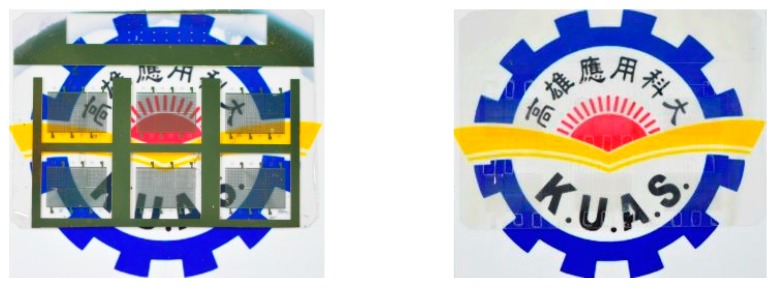
CMUTs with gold (**left**) and silver nanowire (**right**) top electrodes.

**Figure 8:**
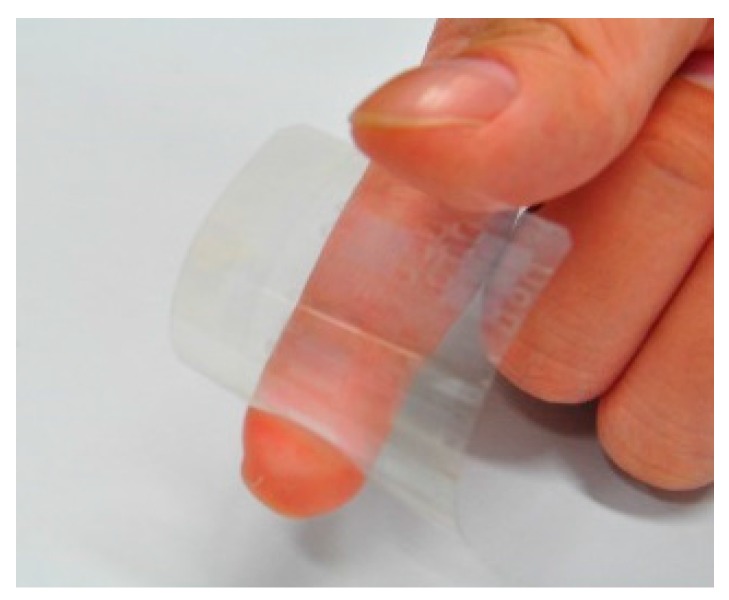
A transparent flexible CMUT.

A transparent flexible CMUT was successfully fabricated using the proposed roll-lamination techniques. Figure 7 presents non-transparent (left image) and transparent (right image) CMUTs with gold and silver nanowire top electrodes. Figure 8 shows a transparent CMUT under deflection. The performance characteristics of these two CMUTs were tested and compared.

## 4. Discussion

### 4.1. Roll-Lamination Fabrication

Fabricating a flexible CMUT through roll-lamination techniques involves two critical steps: (1) preparing vibrating membranes on a PET release layer, and (2) laminating the membranes onto the sidewall. The SU-8 vibrating membranes should be prepared on a PET release layer with a baking temperature below 100 °C. If the temperature is higher than 120 °C, membrane deformation occurs on the PET release layer. The membrane preparation in step (4) with baking times increasing more than 10 s result in an excessively dry SU-8 photoresist, which causes the membranes to fail to laminate onto the sidewall; this is evident when many bubbles form at the junction between the sidewall and the membrane during lamination. The baking times decreasing more than 10 s lead to an overly wet photoresist, which causes the membranes to detach from the sidewall when the release layer is removed; this is evident when the cavity is exposed or when there are holes on the membranes.

**Figure 9:**
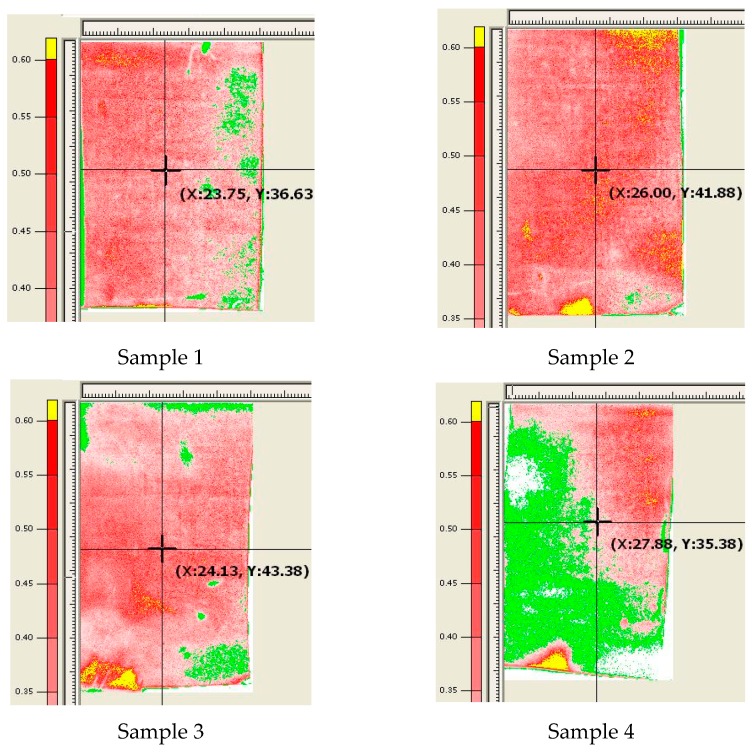
Lamination pressure distribution on four Prescale films. The red color indicates good bonding pressure; the green color with lower pressure leads to bonding failure; the yellow color with higher pressure causes the sidewall deformed. Sample 1 has good lamination on the left half region but not right half region; sample 2 with an uniform and correct pressure is the best among four samples; sample 3 applied uneven pressure leads to good lamination in the central region but not surrounding regions; sample 4 with lower than ideal pressure is the worst.

Successful lamination of vibrating membranes onto the sidewall depends largely on the lamination pressure. When the lamination pressure is below 0.3 MPa, the membranes and sidewall fail to bond; when the lamination pressure is over 0.6 MPa, the sidewall becomes deformed. A Fuji Prescale film is used to measure the lamination pressure and its uniformity on the membranes; this is achieved by placing the film on the PET release layer, observing the color change on the release layer following lamination (yellow: >0.6 MPa; red: 0.3–0.6 MPa; green: <0.3 MPa), and measuring the levels of lamination pressure on the four regions (upper, lower, left, and right) of the release layer. Figure 9 illustrates the lamination pressure distribution on four Prescale films tested by applying various pressure levels. In Sample 1, the lamination pressure has an average of 0.35 MPa and is more uniform on the left half than on the right half; the membranes on the right half region indicated by the green color could not be laminated onto the sidewall. In Sample 2, the lamination pressure was uniform with an average of 0.39 MPa across all the regions; thus, this sample represented the highest success rate among the four samples. In Sample 3, the lamination pressure has an average of 0.4 MPa and the central region has better lamination compared with the surrounding regions. In Sample 4, the lamination pressure was not uniform with an average less than 0.31 MPa; thus, this sample represented the lowest success rate. Note that the best lamination pressure is between 0.35 and 0.55 MPa.

### 4.2. Transparent Electrodes

Three transparent electrode materials, ITO, AZO, and silver nanowire, were used to prepare the CMUT top electrodes to replace the gold top electrode. The ITO top electrode was prepared through vacuum deposition at a vacuum level of 10^−5^ Torr and with a deposition rate of 0.8 Å/s, deposition thickness of approximately 200 nm, and sheet resistance of 160–200 Ω/sq using a four-point probe. The AZO top electrode was prepared through magnetron sputtering at a vacuum level of 10^−3^ Torr and with a deposition rate of 0.5 Å/s, deposition thickness of approximately 200 nm, and sheet resistance of 230–270 Ω/sq. The conductivities of the ITO and AZO electrode cannot meet the conductivity of the gold electrode but reach the normal quality of a transparent conducting film. The silver nanowire electrodes were fabricated by Material and Chemical Laboratories of the Industrial Technology Research Institute, Hsinchu, Taiwan.

**Figure 10:**
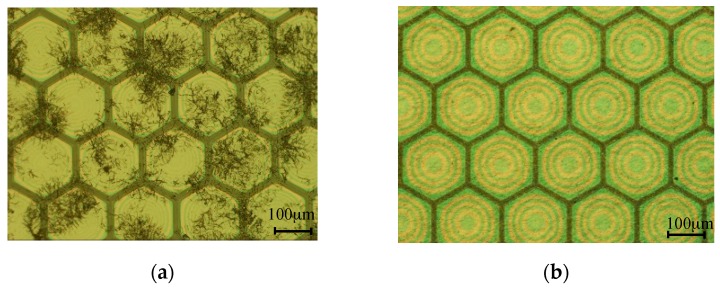
(**a**) Poorly distributed silver nanowire electrode coated on untreated SU-8 membranes; (**b**) Well-distributed and transparent silver nanowire electrode coated on treated SU-8 membranes.

The SU-8 membrane surface should be modified before coating a silver nanowire electrode. The hydrophobicity of the SU-8 membranes causes unevenly distributed silver nanowire paste, so the electrodes have low transmittance and conductivity. The SU-8 membranes were subject to a corona discharge treatment and the contact angle between the SU-8 and silver nanowire paste was dropped from 66° to 22°. The silver nanowire electrodes were prepared through three different fabrication methods: spin coating, spray coating, and dip coating. The silver paste has a nanowire length of 5–25 μm and nanowire diameter of 25–45 nm. The 200 nm thick silver nanowire electrode has a sheet resistance up to the 60 Ω/sq similar to the gold electrode. To achieve the same sheet resistance the dip coating was processed once, spin coating twice, and spray coating four times. The transparency of the electrode drops quickly with more processes so dip coating with the highest transmittance is the best choice. Figure 10a shows a poorly distributed silver nanowire electrode coated on untreated SU-8 membranes. Figure 10b displays a well-distributed and transparent silver nanowire electrode coated on treated SU-8 membranes.

**Figure 11:**
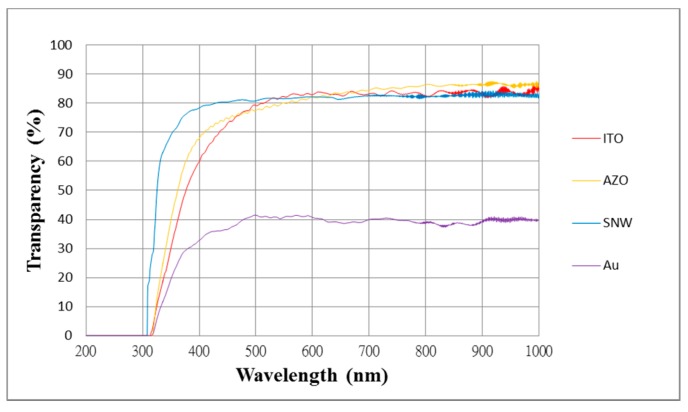
Transmittance measurements of CMUTs with gold, ITO, aluminum-doped zinc oxide (AZO), and silver nanowire top electrodes.

The transmittance of four CMUTs with gold, ITO, AZO, and silver nanowire top electrodes was measured using a Shimadzu UV-1800 spectrometer (Shimadzu Corporation, Kyoto, Japan) in the visible wavelength range, 380–780 nm. The transmittance levels of the gold, ITO, AZO, and silver nanowire top electrodes were 39.7%, 83.2%, 84.1%, and 84.2%, respectively. The CMUTs with ITO, AZO, and silver nanowire electrodes all exceed the 80% transmittance threshold for transparent conducting films. Figure 11 shows the transmittance measurements of the CMUTs with different top electrodes.

**Figure 12:**
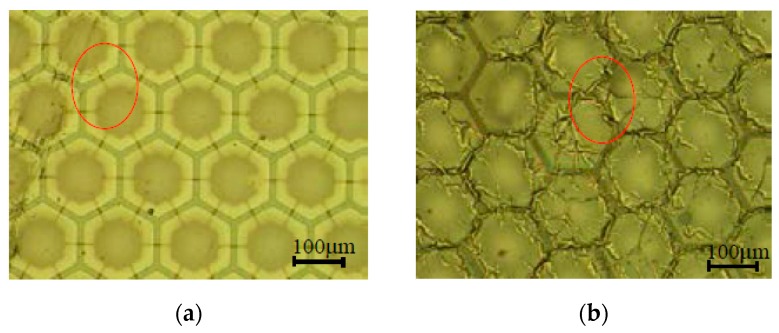
(**a**) Cracks on the ITO top electrode after the pulse-echo test; (**b**) Cracks on the AZO top electrode after the pulse-echo test.

The CMUTs with transparent top electrodes were tested by performing pulse-echo experiments under AC 300 V and DC 100 V bias voltage conditions. The CMUTs with ITO and AZO top electrodes malfunctioned after transmitting ultrasonic signals for less than five minutes. Observation through an optical microscope revealed cracks on the electrodes, which were caused by the vibrating membrane. The cracks of the ITO and AZO top electrodes are clearly shown in Figure 12a,b. There was no improvement with the coating protecting layer. No prior study has investigated the behaviors of ITO and AZO top electrodes under ultrasonic vibration operations, although several studies have reported the increased resistivity of electrodes caused by cyclic bending [28,29,30,31,32,33,34]. However, the resistivity of the silver nanowire electrode reported no change in sheet resistance when flexed over 1000 cycles [35]; thus, the silver nanowire was further investigated as the top electrode for the proposed CMUT.

**Figure 13:**
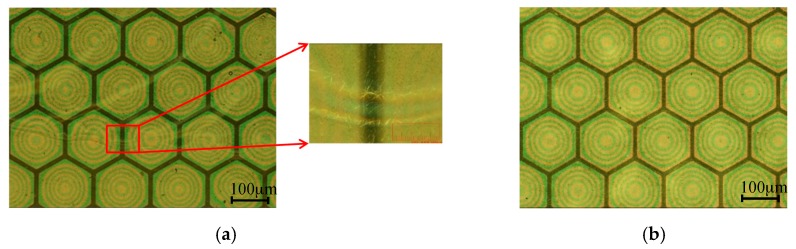
(**a**) Surface warpage observed on a protecting layer after the pulse-echo test; (**b**) No warpage observed on a 1 μm SU-8 protecting layer after the pulse-echo test.

The silver nanowire electrode should be coated with an appropriate protecting layer to prevent the vibration of the membrane from peeling off silver nanowires. The CMUTs with silver nanowire top electrodes were also tested by performing pulse-echo experiments under AC 300 V and DC 100 V bias voltage conditions for a period from 4 to 24 h more than twenty times. When the electrode was first coated with a 200 nm thick protecting layer, warpages occurred on the protecting layer and silver nanowires were peeled off after a pulse-echo experiment for 4 h as shown in Figure 13a. The warpages caused an open circuit of the CMUT. In this study, a 200 nm thick silver nanowire transparent electrode was fabricated on a 4 µm thick SU-8 membrane and was subsequently coated with a 1 µm thick SU-8 protecting layer. There is no warpage of the protecting layer or peeling of the silver nanowire after the pulse-echo test for 24 h as shown in Figure 13b.

Table 2 summaries the performances of the CMUTs with gold, ITO, AZO, and silver nanowire top electrodes. The CMUTs with ITO and AZO top electrodes can be bent or flexed but were damaged after the pulse-echo test for less than five minutes. Thus, both electrodes cannot be used in ultrasound applications. However, the silver nanowire electrode with the SU-8 protecting layer is the best choice for the transparent flexible CMUTs.

### 4.3. Membrane Swelling

**Figure 14:**
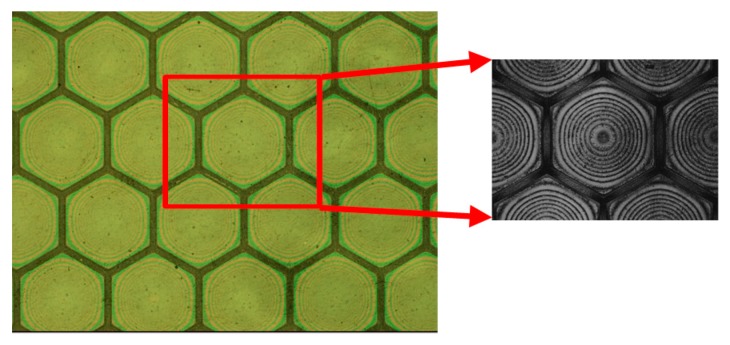
Newton’s rings on the membranes increase in number after 24 h of operation.

**Figure 15:**
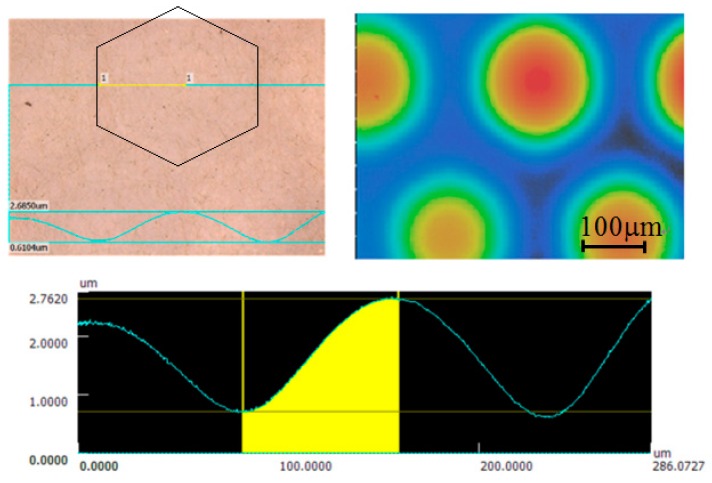
Swelling of the transparent CMUT membranes after 24 h of operation.

The SU-8 vibrating membranes of the CMUTs swelled initially after fabrication and further expanded after extended use. The swelling behavior is influenced by the baking procedure in photoresist development process and internal stresses in the SU-8 film [36]. The swelling height was obtained by calculating the number of Newton’s rings on the membrane and by using a Keyence VK-X250 3D laser microscope. There were six Newton’s rings observed on the transparent CMUT membranes after fabrication, and this increased to 14 rings after over 24 h of continuous operation, as shown in Figure 14. In addition, the 3D microscope revealed that the swelling height increased from 0.9 to 2.0 µm, as shown in Figure 15. The membrane swelling enlarged the distance between the top and bottom electrodes and caused a reduction in ultrasonic signals of the CMUT.

## 5. CMUT Characteristics Measurement

**Figure 16:**
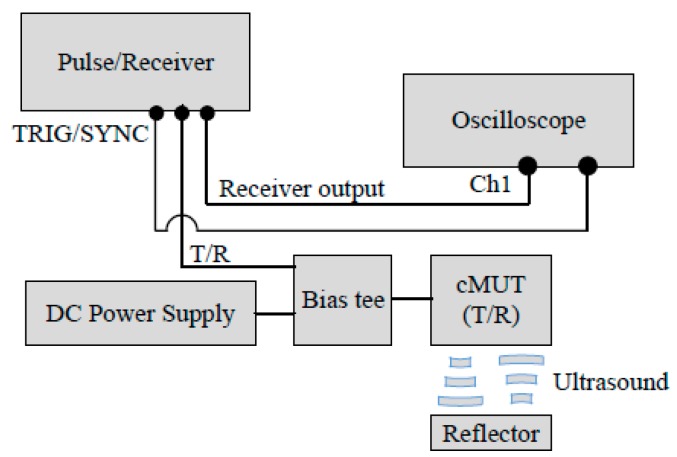
CMUT pulse-echo test framework.

The performance of the transparent CMUT with the silver nanowire top electrode was investigated using pulse-echo tests and compared to the CMUT with the gold top electrode. Figure 16 illustrates the framework of the CMUT pulse-echo experiment. The tests included operating voltage conditions, time and frequency responses under a 24 h operation, detection distance versus time of flight, and maximum detection distances with CMUTs mounted on flat and curved surfaces.

**Figure 17:**
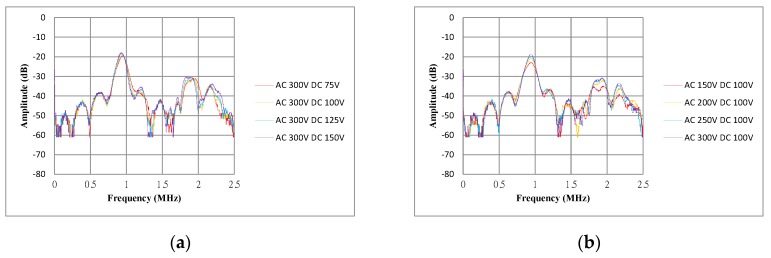
(**a**) Spectra of received signals of the transparent CMUT under AC 300 V and various DC bias voltage; (**b**) Spectra of received signals of the transparent CMUT under DC 100 V and various AC pulse voltage.

The operating voltage conditions were tested on the transparent CMUT by detecting a flat target at a distance of 10 mm at various AC pulse voltages and DC bias voltages. The AC pulse voltage was increased from 150 to 300 V in 50 V increments, whereas the DC bias voltage was increased from 75 to 150 V in 25 V increments. The upper limits of both the AC and DC voltages were specified to prevent transducer burnout. Figure 17 shows the spectra of the received signals of the transparent CMUT at different operating voltage conditions. The CMUT can detect the maximum distance at the AC 300 V, DC 100 V condition and had a resonance frequency of 880 kHz with a maximum amplitude of −18.6 dB.

**Figure 18:**
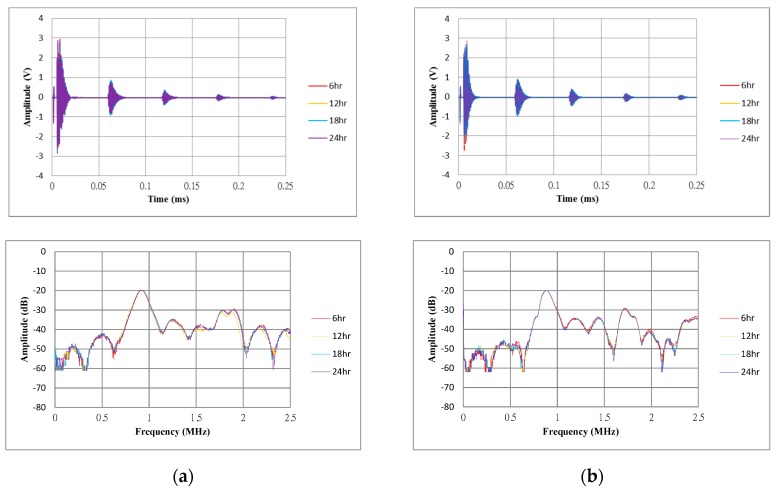
(**a**) Time and frequency responses of the CMUTs with gold electrodes; (**b**) Time and frequency responses of the CMUTs with silver nanowire electrodes.

Figure 18a,b present the time and frequency responses of both gold and silver nanowire CMUTs operating for 24 h at the AC 300 V, DC 100 V condition by detecting a flat target at a distance of 10 mm. The maximum amplitudes of the first reflection signal for the gold and silver nanowire CMUT were 860 and 960 mV, respectively. The natural frequencies were 880 kHz for both CMUTs. There is no change was observed during 24 h of operation.

**Figure 19:**
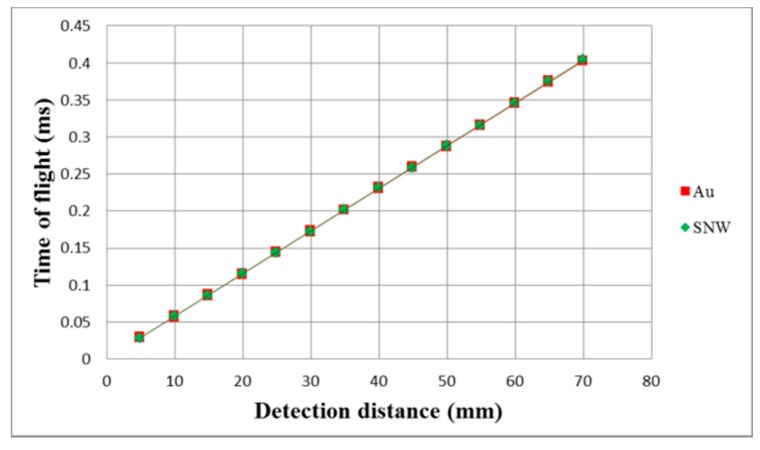
Detection distance relative to time of flight.

Figure 19 shows the detection distance versus time of flight experiments for both gold and silver nanowire CMUTs. The experiments were conducted at room temperature 25 °C with the target distances ranging from 5 to 70 mm in 5 mm increments. Each measurement was repeated three times. The propagation speeds for the gold and silver nanowire CMUTs were 347.5 m/s and 346.5 m/s, respectively. The linearity errors of the displacement were 0.136 mm and 0.141 mm.

**Figure 20:**
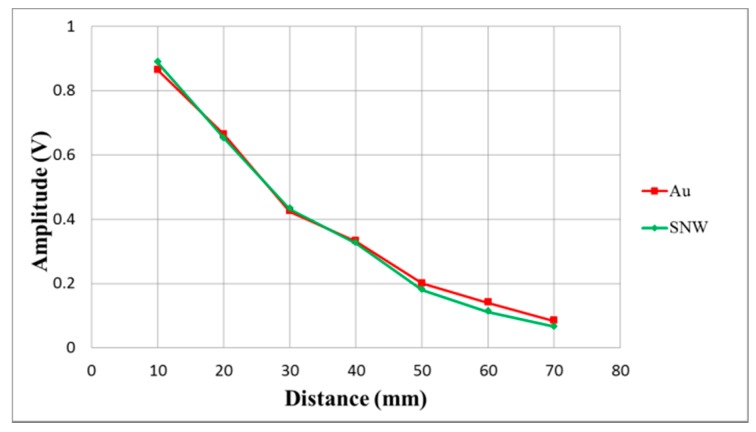
Reflection signals at different detection distances.

Figure 20 illustrates detection distance versus reflection signal experiments for both gold and silver nanowire CMUTs. The maximum detection distances were 70 mm for both CMUTs. The maximum reflection signals for gold and silver nanowire CMUTs were 864 mV and 888 mV at a 10 mm detection distance, and decreased to 84 mV and 66 mV at 70 mm. There were only slight differences observed for both CMUTs.

**Figure 21:**
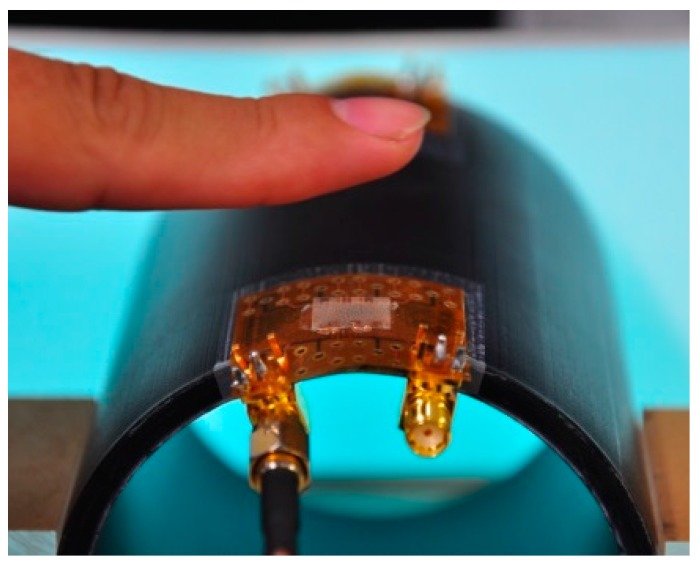
A transparent CMUT mounted on a 40 mm curvature radius surface to detect a finger.

**Figure 22:**
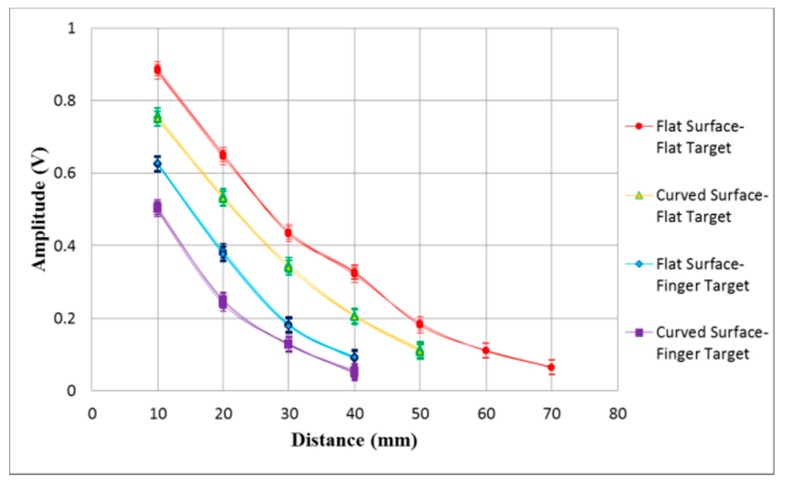
Distance measurement signal of the transparent CMUTs mounted on flat and curved surfaces to detect a flat target and a finger.

The transparent flexible CMUTs were studied on both flat and curved surfaces to determine the maximum detection distances for curved displays and wearable electronics applications. The CMUTs were mounted on a cylinder with a 40 mm curvature radius to detect flat targets and a finger. The maximum detection distances were determined in 10 mm increments until the reflection signal amplitude diminished to 50 mV. Figure 21 shows a photograph of a finger being detected by the silver nanowire CMUT on a curved surface. Figure 22 presents the experimental results of the CMUT on both flat and curved surfaces to detect a flat target and a finger. Table 3 summaries the reflection signal amplitudes at a distance of 10 mm and the maximum detection distances under different operating conditions. The CMUT on the flat surface obtained a maximum detection distance of 70 mm and 50 mm from the flat target and finger, respectively. The CMUT on the curved surface obtained a maximum detection distance of 50 mm and 40 mm from the flat object and finger.

## 6. Conclusions

This paper presents a novel transparent flexible CMUT made by low temperature roll-lamination fabrication processes. Three transparent electrode materials, ITO, AZO, and silver nanowire, were applied for the CMUT top electrodes. This research found that ultrasonic vibration caused cracks of the ITO and AZO transparent electrodes on the vibrating membrane. The CMUT adopted silver nanowire transparent electrodes that can continuously operate over 24 h without any performance deterioration. The CMUT on the flat surfaces can detect a flat target at distances up to 70 mm with a linearity error of 0.141 mm. The transducer, when operated on a curved surface with a 40 mm curvature radius, detected a finger at a distance of 40 mm. The research compared the performances of two CMUTs using a gold non-transparent electrode and a silver nanowire transparent electrode. Except for transmittance there is no significant difference between the two CMUTs in terms of sensor characteristics and operating conditions. The transparent flexible CMUT can be easily integrated with a curved display and wearable electronics for non-contacting control at short distances and provides more advanced human-machine interaction than existing touch panels.

## Figures and Tables

**Table 1:** Transparent CMUT dimensions.

Membrane diameter	A	140 µm	Membrane thickness	D	5 µm
Silver nanowire (SNW) electrode diameter	B	160 µm	SNW electrode thickness	E	0.2 µm
Sidewall height	F	2 µm	Sidewall width	C	10 µm
Indium Tin Oxide (ITO) electrode thickness	G	0.2 µm	Polyethylene terephthalate (PET) thickness	H	125 µm

**Table 2:** Performances of CMUTs with gold, ITO, AZO, and silver nanowire top electrodes.

	Gold	ITO	AZO	SNW
Thickness (nm)	~150	~200	~200	~200
Sheet resistance (Ω/sq)	10–60	160–200	230–270	10–60
Transmittance (%)	39.4	83.2	84.1	84.2
Color	Gold	Transparent	Transparent	Transparent
Flexibility	Good	OK	OK	Good
Ultrasound Application	Good	Damaged	Damaged	Good

**Table 3:** Reflection signals and maximum detection distances of transparent CMUT obtained on flat and curved surfaces for flat target and finger.

Mounted Surface	Target Object	Reflection Signal at 10 mm (mV)	Maximum Detection Distance (mm)
Flat	Flat	888	70
Flat	Finger	624	50
Curved	Flat	762	50
Curved	Finger	506	40
